# Snail Involves in the Transforming Growth Factor β1-Mediated Epithelial-Mesenchymal Transition of Retinal Pigment Epithelial Cells

**DOI:** 10.1371/journal.pone.0023322

**Published:** 2011-08-10

**Authors:** Hui Li, Hongwei Wang, Fang Wang, Qing Gu, Xun Xu

**Affiliations:** 1 Department of Ophthalmology, Shanghai First People's Hospital, Affiliate of Shanghai Jiaotong University, Shanghai, China; 2 Department of Medicine, University of Chicago, Chicago, Illinois, United States of America; 3 Department of Ophthalmology, Shanghai Tenth People's Hospital, Affiliate of Tongji University, Shanghai, China; University of Sao Paulo – USP, Brazil

## Abstract

**Background:**

The proliferation of retinal pigment epithelium (RPE) cells resulting from an epithelial-mesenchymal transition (EMT) plays a key role in proliferative vitreoretinopathy (PVR), which leads to complex retinal detachment and the loss of vision. Genes of Snail family encode the zinc finger transcription factors that have been reported to be essential in EMT during embryonic development and cancer metastasis. However, the function of Snail in RPE cells undergoing EMT is largely unknown.

**Principal Findings:**

Transforming growth factor beta(TGF-β)-1 resulted in EMT in human RPE cells (ARPE-19), which was characterized by the expected decrease in E-cadherin and Zona occludin-1(ZO-1) expression, and the increase in fibronectin and α-smooth muscle actin (α-SMA) expression, as well as the associated increase of Snail expression at both mRNA and protein levels. Furthermore, TGF-β1 treatment caused a significant change in ARPE-19 cells morphology, with transition from a typical epithelial morphology to mesenchymal spindle-shaped. More interestingly, Snail silencing significantly attenuated TGF-β1-induced EMT in ARPE-19 cells by decreasing the mesenchymal markers fibronectin and a-SMA and increasing the epithelial marker E-cadherin and ZO-1. Snail knockdown could effectively suppress ARPE-19 cell migration. Finally, Snail was activated in epiretinal membranes from PVR patients. Taken together, Snail plays very important roles in TGF-β-1-induced EMT in human RPE cells and may contribute to the development of PVR.

**Significance:**

Snail transcription factor plays a critical role in TGF-β1-induced EMT in human RPE cells, which provides deep insight into the pathogenesis of human PVR disease. The specific inhibition of Snail may provide a new approach to treat and prevent PVR.

## Introduction

Proliferative vitreoretinopathy (PVR), a scarring process that develops with some retinal detachments (RDs), is the most common cause of surgical failure in the rhegmatogenous RD treatment [1]. PVR is a dynamic process which is characterized by the formation of fibrotic tissue both on the detached retina. Fibrotic tissue on the detached retina reduces the flexibility of retina and may potentially make it difficult to reattach to the retina [1], [2]. The retinal pigment epithelium (RPE) cells are main contributor to the development of fibrotic tissue on the retina. RPE cells contain various other cell types including glial cells that are involved in the fibrotic reaction of the detached retina [1]–[3]. Adult retinal pigment epithelial cells are quiescent, differentiated, and reside in the Go phase of the cell cycle [4]. However, with the development of retinal break and detachment and alteration of the Blood-retina Barrier, RPE cells are exposed to a variety of cytokines, growth factors containing in serum. RPE cells then undergo epithelial-mesenchymal transition (EMT) and form fibroblast-like cells, and produce extracellular matrix components participating in the fibrotic tissue formation on the detached retina [5].

EMT is an orchestrated series of events, in which differentiated epithelial cells undergo phenotypic transition to mesenchymal cells, often fibroblasts and myofibroblasts [6]. During EMT, the epithelial cells lose intracellular junctions causing dissociation from surrounding cells, acquire mesenchymal-like characteristics and become able to migrate away from the original location [7]. This important process was initially recognized during embryonic development and has more recently been implicated in tumor progression and organ fibrosis [6]–[8]. The current evidence suggests that kidney proximal tubule epithelial cells undergo EMT to induce interstitial fibrosis in progressive renal disease [8], [9]. In the fibrotic kidney, about 36% new fibroblasts arise from tubular epithelial cells [8]. EMT also contributes to the development of ocular fibrotic disorder. During the formation of anterior polar cataracts and posterior capsular cataracts, lens epithelial cells trans-differentiate and proliferate into plagues of large spindle-shaped cells, or myofibroblasts through EMT [10]. EMT can be triggered by different signalling molecules, such as transforming growth factor beta (TGF-β), epidermal growth factor (EGF), fibroblast growth factor (FGF), hepatocyte growth factor (HGF), bone morphogenetic proteins (BMPs) and WNTs and Notch [11]. TGF-β-mediated EMT has been observed in a variety of cell types, including lens epithelial cells [12].

A wide array of transcription factors, including Snail, Slug (SNAI2), δEF1 (ZEB1), SIP1 and Twist, are involved in regulating EMT [13]. Snail, a zinc-finger transcription factor, has been characterized as a key EMT regulator [11]. Studies showed that Snail bind to specific DNA sequences called E-boxes in the promoter of E-cadherin gene and repress transcription of E-cadherin [14], [15]. Therefore, down-regulation of the cell-cell adhesion protein E-cadherin was considered as a hallmark of EMT. Knockout mice deficient for Snail die at gastrulation because they fail to undergo a complete EMT process and form an abnormal mesodermal layer that maintains E-cadherin expression [16]. In some epithelial tumor cell lines, Snail-regulated EMT promotes cell motility and invasion [11], [13]. An inverse correlation between E-cadherin and Snail expression has been noted in cultured epithelial lines established from breast cancer, pancreatic carcinoma and colon cancer [14]. Silencing of Snail by stable RNA interference in epithelial Madin Darby canine kidney (MDCK)-Snail cells attenuated the complete EMT, which associates with the up-regulation of E-cadherin, down-regulation of mesenchymal markers and inhibition of invasion [17].

Despite the regulation of Snail transcription factor in EMT during cancer progressive and some fibrotic disorders has been extensively studied, the role of Snail in ocular fibrotic diseases, especially PVR, is rarely defined. In the present study, we first showed that Snail transcription factor plays an essential role in TGF-β1-mediated EMT in human RPE cells. We also report the presence of pathological Snail expression in epiretinal membranes from PVR patients. These findings may help us further understand the molecular mechanisms behind the pathogenesis of PVR, this might provide opportunities to prevent and treat human PVR effectively.

## Results

### Endogenous Snail expression in ARPE-19 cells

**Figure 1:**
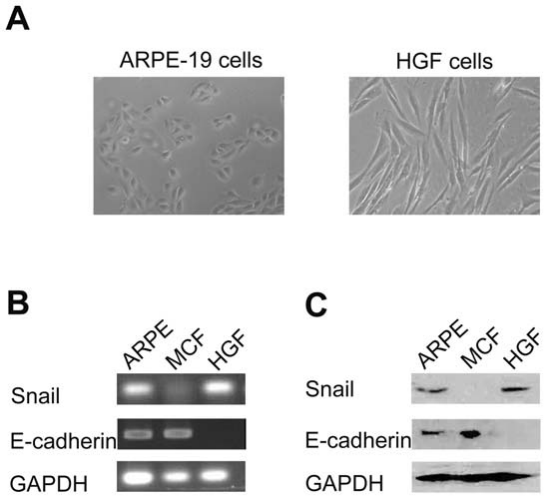
The correlation between endogenous expression of Snail and E-cadherin in ARPE-19 cells. (A) Compared to HGF cells, ARPE-19 cells showed the typical epithelial morphology. Original magnifications 100×. RT-PCR (B) and Western blot (C) analysis of Snail and E-cadherin in ARPE-19, MCF-7 and HGF cells at the mRNA and protein levels respectively. MCF-7 cells were used as a positive control for E-cadherin expression and HGF cells were used as a positive control for Snail expression.

The general morphology of the human RPE cells (ARPE-19) was observed. As showed in Fig. 1A, ARPE-19 cells demonstrate an organized epithelial morphology and form a cobblestone-like monolayer. However, HGF cells show typical mesenchymal morphology. There is an reverse relationship between expression of Snail and epithelial marker E-cadherin in some epithelial tumor cell lines cells [14], [15]. Hence, we next investigated endogenous expression of Snail in ARPE-19 cells and the possible correlation between the expression of Snail and E-cadherin. RT-PCR revealed a low level of endogenous Snail mRNA in ARPE-19 cells (Fig. 1B). Western blot confirmed the above result at protein level (Fig. 1C). ARPE-19 cells expressed the epithelial marker E-cadherin at the mRNA and protein levels (Fig. 1B and C). Interestingly, the expression of Snail is reversely associated with E-cadherin expression in MCF and HGF cells respectively (Fig. 1B and C).

### EMT induced by TGF-β1 in ARPE-19 epithelial cells

**Figure 2:**
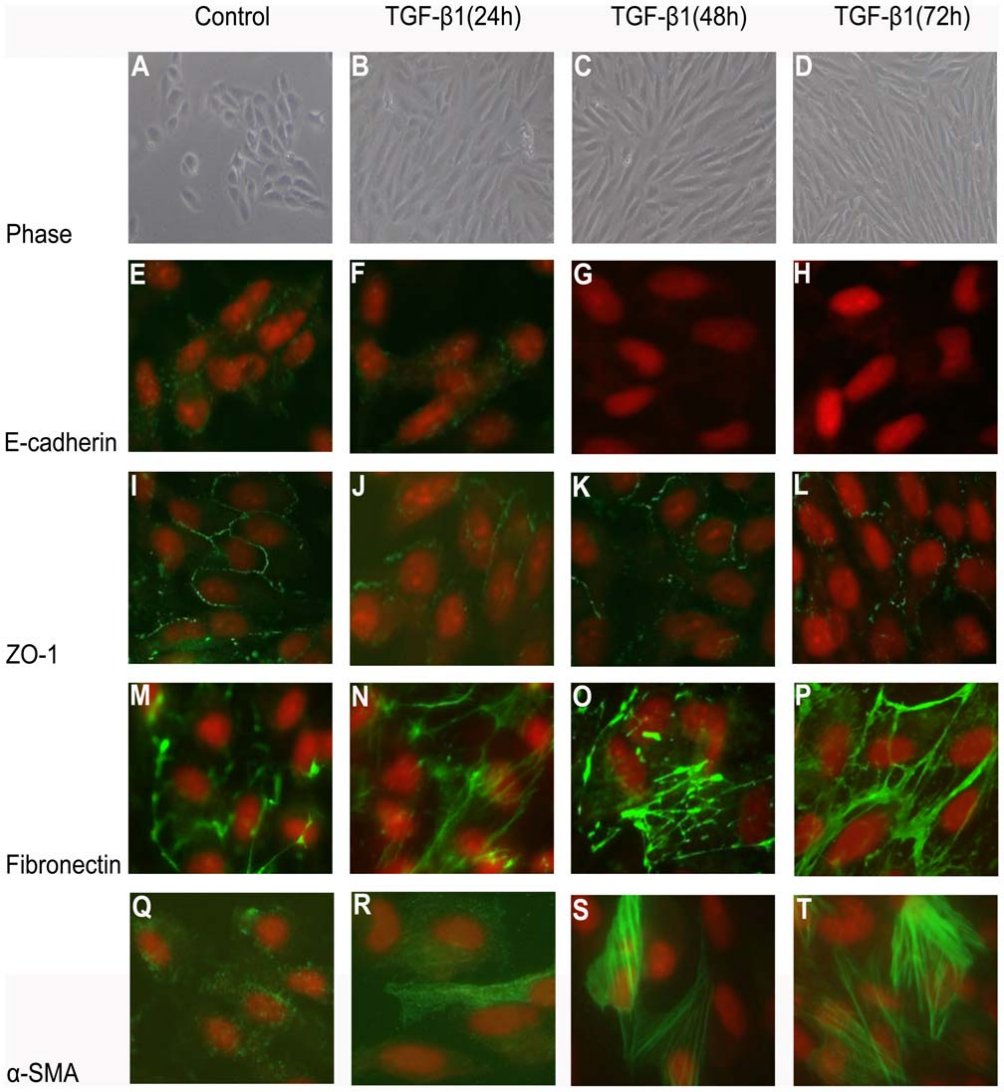
EMT of ARPE-19 cells induced by TGF-β1. ARPE-19 cells were grown on glass coverslips for 24 hr, starved for 24 hr, and then incubated for 24 hr, 48 hr, 72 hr with 10 ng/ml TGF-β1. Compared with control cells (A, E, I, M, Q), stimulated cells displayed an altered mesenchymal morphology by phase-contrast microscopy (B, C, D), with decreasing expression of E-cadherin (F, G, H) and ZO-1 (J, K, L) and an increasing expression of fibronectin (N, O, P) and α-SMA (R, S, T) by immunofluorescence microscopy. The green signal represents the staining of corresponding protein, and the red signal represents the nuclei staining by DAPI. Original magnifications 100× (A–D); 400× (E–T).

Although various kinds of growth factor and cytokine orchestrate the process of EMT, TGF-β1 is believed to play a major role in this process [5]. During EMT, the epithelial markers E-cadherin and zona occludin-1 (ZO-1) are down-regulated, whereas the mesenchymal markers fibronectin and α-smooth muscle actin (α-SMA) are up-regulated [16], [17]. Therefore, the EMT of ARPE-19 cells was observed after stimulation with TGF-β1. Stimulation of ARPE-19 cell monolayer with 10 ng/ml TGF-β1 resulted in a significant change in cell morphology, as demonstrated by phase-contrast microscopy, with transition from a typical cobblestone morphology to mesenchymal spindle-shaped and fusiform features (Fig. 2A, B, C and D). These morphological changes were associated with the loss of epithelial characteristics E-cadherin (Fig. 2E, F, G and H) and ZO-1 (Fig. 2I, J, K and L) and with the acquisition of some mesenchymal characteristics, including an increase in matrix protein fibronectin (Fig. 2M, N, O and P) and α-SMA expression (Fig. 2Q, R, S and T). α- SMA is considered a marker of myofibroblast, which is a cell type found in EMT. The acquisition of a fibroblastic morphology and mesenchymal markers suggested that ARPE-19 cells had undergone an EMT after treated by TGF-β1.

### TGF-β1 stimulation induces up-regulation of Snail

**Figure 3:**
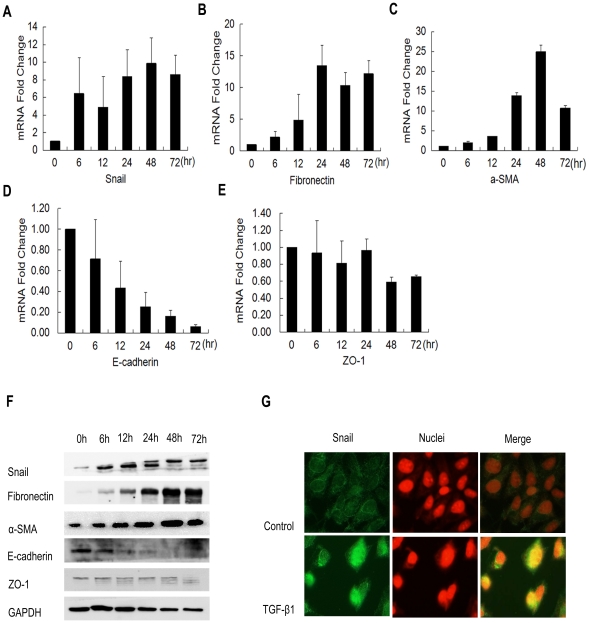
TGF-β1 increased Snail expression in cultured ARPE-19 cells. Total RNA was extracted from ARPE-19 cells at the time points as indicated after TGF-β1 (10 ng/ml) treatment. (A) The increasing expression of Snail mRNA was time-dependent. The increasing expression of mesenchymal markers fibronectin (B) and α-SMA (C) and the decreasing expression of epithelial markers E-cadherin (D) and ZO-1 (E). Values were normalized to GAPDH mRNA levels and represent fold change as compared to untreated cells. Data shown as mean ± SD of two independent experiments each performed in triplicate. (F) Western blot analysis of Snail, fibronectin, α-SMA, E-cadherin and ZO-1 with proteins extracted from ARPE-19 cells treated with 10 ng/ml TGF-β1 at differential time points. (G) Imunofluorescence analysis and subcellular localization of Snail (green) in ARPE-19 cells treated with 10 ng/ml of TGF-β1 for 48 hr. Nuclei were stained with DAPI (red). Original magnifications 400×.

Zinc-finger transcription factor Snail plays an essential role in regulating EMT during tumor progression and organ fibrosis [11], [13], [17]. Because of the association of Snail with EMT, we then investigated whether expression of Snail was up-regulated by TGF-β1 in ARPE-19 cells. Compared to untreated cells, TGF-β1 increased Snail mRNA expression 6 hr after treatment, and reached the highest level at 48 hr (Fig. 3A). The inducing effects of TGF-β1 on Snail protein levels were confirmed by Western blotting (Fig. 3F). In addition, we confirmed the increasing expression of mesenchymal markers fibronectin and α-SMA (Fig. 3B, C and F), and the decreasing expression of epithelial markers E-cadherin and ZO-1 (Fig. 3D, E and F) at mRNA and protein levles.

Control of the nuclear localization of transcription factors is an important process in response to external stimuli because transcription factors can not function until they translocate to the nucleus [18]. As shown in Fig. 3G, we found that TGF-β1 treatment for 48 hr induced Snail protein translocation to the nucleus. By contrast, in untreated ARPE-19 cells, almost no Snail protein was detected in the nucleus. Based on these observations, we concluded that Snail will be activated after TGF-β1 stimulation which resulted in a nuclear translocation of Snail in ARPE-19 cells.

### Suppression of Snail attenuates TGF-β1-induced EMT

**Figure 4:**
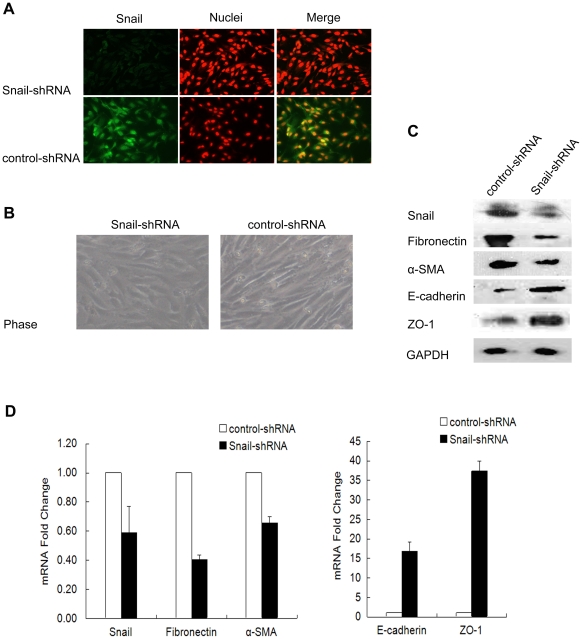
Snail silencing attenuated TGF-β1 induced EMT. (A) Immunofluorescence staining of Snail in Snail-shRNA or control-shRNA transfected cells which were starved for 24 hr in serum-free media and treated with 10 ng/ml of TGF-β1 for 48 hr. The green signal represents the staining of Snail protein, the red signal represents the nuclei staining by DAPI. (B) Photomicrographs of Snail-shRNA or control-shRNA transfected ARPE-19 cells are showing different cell morphologies. Cells transfected with Snail-shRNA or control-shRNA plasmid were starved for 24 hr in serum-free media and treated with 10 ng/ml of TGF-β1 for 48 hr. The expression of Snail, fibronectin, a-SMA, E-cadherin and ZO-1 were analyzed by Western blot (C) and real-time PCR (D). Values represent fold change as compared to control cells. Data shown as mean ± SD of two independent experiments each performed in triplicate. Original magnifications 400× (A); 100× (B).

Since TGF-β1 stimulation can induce EMT and up-regulate Snail expression in ARPE-19 cells, we speculated that snail might be involved in EMT of ARPE-19 cells. To understand the specific biological functions that Snail exerts during TGF-β1 induced EMT, we knocked down Snail in ARPE-19 cells transfected with a Snail-targeting siRNA-expressing plasmid. The plasmid pSuper-Snail-shRNA contains a small hairpin RNA (shRNA) transcription unit which can be processed into Snail-targeting siRNA in cells [19]. Firstly, the expression of Snail protein in nucleus was observed after 48 hr treatment of TGF-β1 by immunofluoresence. As shown in Fig. 4A, the expression of Snail in pSuper-Snail-shRNA transfected cells was down-regulated significantly, compared with pSuper-control transfected cells. We then observed the morphological changes in TGF-β1-induced ARPE-19 cells after Snail silencing. Compared with pSuper-control transfected cells, pSuper-Snail-shRNA transfected cells showed epithelial morphology (Fig. 4B). These results showed that Snail levels were decreased in pSuper-Snail-shRNA transfected cells and silencing of Snail protein reversed TGF-β1-induced morphological transition. We next tested the molecular mechanisms underlying the process. As shown in Fig. 4C and 4D, the expression of Snail was detected after TGF-β1 stimulation for 48 hr in cells transfected with pSuper-Snail-shRNA or with pSuper-control. However, the expression of Snail in pSuper-Snail-shRNA transfected cells significantly decreased compared with control cells. These results showed that shRNA against Snail could reduce the expression of Snail efficiently. At the same time, down-regulation of Snail resulted in the decrease of the mesenchymal markers fibronectin and a-SMA and the increase of the epithelial markers E-cadherin and ZO-1 (Fig. 4C and D).

### Snail silencing effectively suppress migration of ARPE-19 cells

**Figure 5:**
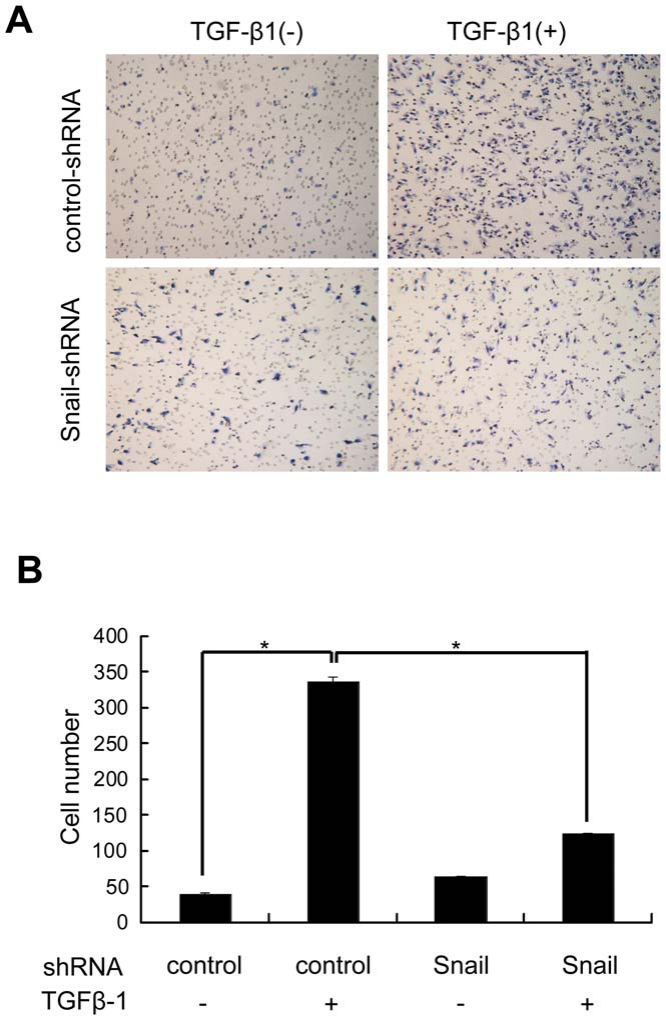
Migration after Snail knockdown in ARPE19 cells with or without TGF-β1 treatment. (A) Cells transfected with control-shRNA or Snail-shRNA were allowed to migrate transwell chambers for 18 hr in the presence or absence of TGF-β1. After 18 hr, the migrated cells were fixed, stained, and photographed. (B) The number of migrated cells. Data shown represent the average of three independent experiments. *P<0.05, compared with control-shRNA, TGF-β1(−) samples and control-shRNA, TGF-β1(+) samples.

EMT can increase the cell motility. To determine the functional changes in ARPE-19 cells behavior that occurred following suppression of Snail, we used Transwell chamber assays to gauge the migratory ability of Snail-knockdown cells in the presence or absence of TGF-β1. Snail expression was blocked by shRNA, and then equal numbers of control-shRNA or Snail-shRNA transfected ARPE-19 cells were added to transwell inserts and allowed to migrate in the presence or absence of TGF-β1 (10 ng/ml). TGF-β1 promoted an obviously increase in migration of control cells, whereas this process was significantly reduced by Snail knockdown in cells transfected with Snail-shRNA (Fig. 5A and B).

### Expression of Snail protein in PVR epiretinal membranes

**Figure 6:**
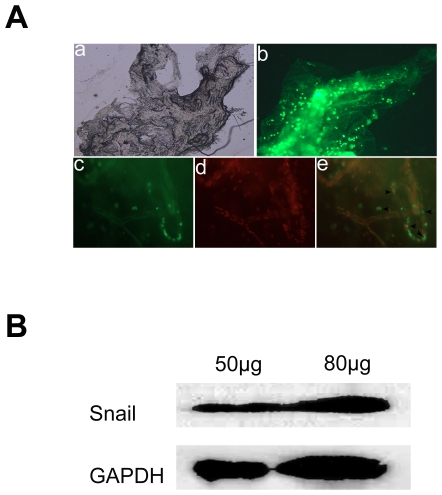
Snail distribution and protein levels in PVR membranes. (A) Immunofluorescent staining of Snail (green) in epiretinal membranes. Nuclei were stained with DAPI (red). Arrowheads indicated colocalization of Snail expression and nuclei. Original magnifications 50× (a); 200× (b–e). (B) 50 µg and 80 µg total protein extracted from PVR epiretinal membranes were detected by Western blot. GAPDH was used as internal control.

To assess whether Snail involved in the pathological process of PVR, we investigated Snail protein distribution and expression in the PVR epiretinal membranes. Immunofluorescent staining detected the presence of Snail in epiretinal membranes from PVR patients (Fig. 6A). Snail was primarily expressed in the nucleus. No fluorescent signals were detected in negative controls (data not shown). Western blot further confirmed Snail protein levels observed in the immunostaining (Fig. 6B).

## Discussion

Epithelial to mesenchymal transition is an essential morphological conversion occurring during embryonic development, and this process is re-engaged in adults during wound healing, tumor progression and organ fibrosis [20]. PVR, an ocular fibrotic diseases, is a process of fibrocellular proliferation in the vitreous cavity and on both surfaces of the retina that may form contractile epiretinal membranes [1], [2], [21]. The gradual contraction of epiretinal membranes causes a marked distortion of the retinal architecture and eventually results in complex retinal detachments and the loss of vision. RPE cells are the major cell type involved in the pathogenesis of PVR, which results from the EMT, proliferation, and migration of transformed RPE cells to the vitreous[5], [21], [22]. The pathological significance of the EMT in the development of PVR is becoming increasingly recognized. However, the process underlying conversion between RPE cells and (myo)fibroblasts is poorly understood. The present findings directly show, for the first time, that Snail plays an essential role in TGF-β1-mediated EMT in human RPE cells and PVR is accompanied by the activation of Snail expression.

TGF-β1 has been showed to play central roles in initiating EMT in models of metastatic tumour development and in fibrogenesis in progressive kidney disease [23]. Previous study indicated that TGF-β1 was detected in vitreous samples, subretinal fluide and epiretinal membranes which were surgically obtained from patients with PVR [24]. The molecules that cause EMT in RPE cells have not been clearly identified, but it is probable that TGF-β1 participates in this process. Some reports recently identified that TGF-β1 induced RPE cells to express a-SMA protein and undergo fibroblastic-type cytoskeletal rearrangements in vitro [25], [26]. Consistent with the above research, our results showed that RPE cells transited from a typical epithelial morphology to fibroblast-like cells after TGF-β1 treatment, which accompanied by increased expression of a-SMA and another mesenchymal marker fibronectin [17]. Myofibroblasts, characterized by the expression of a-SMA, are the predominant cellular component of PVR epiretinal membranes [27]. Interestingly, TGF-β1 was unable to induce EMT without disrupting the integrity of cell-cell contact, indicating involvement of E-cadherin in TGF-β1-mediated EMT [28]. E-cadherin is the main component of adhesive junctions, which plays an important role in the assembly of junctional complexes and the maintenance of epithelial cell morphology. Down-regulation of E-cadherin has been accepted as a hallmarker of EMT [29]. In the present study, we found that EMT was induced in ARPE-19 cells with decreasing expression of cell-cell junctional proteins E-cadherin and ZO-1, and increasing expression of mesenchymal markers a-SMA and fibronectin after TGF-β1 treatment. Lee et al also observed the loss of ZO-1 in ARPE-19 cell EMT [26].

The E-cadherin suppressor, Snail, has been considered as the key regulator of EMT in normal development and tumor progression [14], [15], [30]. We demonstrated for the first time that Snail transcription factor was up-regulated and subsequently E-cadherin was down-regulated during EMT of ARPE-19 cells. Ohkubo and Ozawa demonstrated that ectopic expression of Snail down-regulated the expression of E-cadherin and ZO-1 [31]. With the increased expression of Snail induced by TGF-β1, the decreasing expression of ZO-1 in ARPE-19cells was also observed in this study. Furthermore, the increasing expression of mesenchymal markers fibronectin and α-SMA were consistent with the up-regulation of Snail. Snail exerts a crucial role in the process of fibroblast activation and myofibroblast transdifferentiation [32]. Snail activation is sufficient to induce EMT and kidney fibrosis in adult transgenic mice and interestingly, Snail is also activated in patients with renal fibrosis [33]. Our data showed the nuclear immunoreactivity of Snail in epiretinal membranes from PVR patients. In agreement with our studies, Abu El-Asrar et al showed that there was a significant correlation between the number of a-SMA-positive myofibroblasts and the number of myofibroblasts expressing Snail in PVR epiretinal membranes [34]. Hence, the activation of Snail may have effects on the formation of epiretinal membranes during human PVR. Transcription factors, which are implicated in the compartmentalization and are synthesized in the cytoplasm, are considered to be imported into the nucleus in order to exert their activity [18]. After 48 hr treatment by TGF-β1, most of the activated Snail protein was detected in the nucleus in ARPE-19 cells. The nuclear immunoreactivity of Snail in myofibroblasts was also confirmed in PVR epiretinal membranes through Immunohistochemical techniques [34]. Snail's activity is regulated by various signaling pathways at multiple stages. TGF-β1 has been shown to regulate Snail expression through Glycogen synthase kinase-3β (GSK-3β) [35], as well as Ras, Smad, MAP kinase and Phosphatidylinositol 3-kinase (PI3K) [36], [37]. Further studies will need to address the signal pathway in detail for TGF-β1-mediated Snail expression during the development of EMT in human RPE cells.

After identifying the relationship between TGF-β1 stimulation and the expression and nuclear translocation of Snail in ARPE-19 cell, we then determined whether Snail was sufficient to induce EMT through knockdown experiments. Down-regulation of Snail by a siRNA-expressing vector decreased the expression of mesenchymal markers fibronectin and α-SMA, indicating that Snail is involved in maintaining the mesenchymal properties of transformed ARPE-19 cells. Simultaneously, our results showed that Snail knockdown dramatically increased the protein levels of E-cadherin and ZO-1, suggesting that Snail is required for suppressing these important cell-cell adhesion molecules. In addition, down-regulation of Snail significantly reduced the ability of ARPE-19 cells migration in response to TGF-β1. These results indicated that there was a functional linkage between Snail expression and TGF-β1-mediateed EMT in RPE cells. However, knockdown of Snail did not reverse the full EMT that induced by TGF-β1 treatment in ARPE-19 cells. Previous studies have demonstrated the function of another zinc-finger transcription factor Slug, a close relative of Snail, in regulating EMT during early development and cancer progression [11], [38]. Stable expression of Slug in MDCK cells leads to a full EMT associated with the complete repression of E-cadherin expression, and increased expression of mesenchymal markers as well as acquisition of a highly migratory behavior [39]. It's worth to investigating whether Snail cooperates with Slug for the regulation of TGF-β1-induced EMT in ARPE-19 cell in further studies.

In conclusion, we showed that induction of EMT in human RPE cells led to up-regulation of Snail expression, and inhibition of Snail limited the development of EMT. Specifically, the expression of Snail was detected in epiretinal membranes from human PVR, and that, this implicates the role of Snail in PVR pathogenesis. These findings demonstrate that Snail transcription factor plays a critical role in TGF-β1-induced EMT in human RPE cells and Snail may be partially responsible for the formation of epiretinal membranes found in patients with PVR. The specific inhibition of Snail may provide a new approach for the prevention and treatment of human PVR.

## Materials and Methods

### Ethics Statement

The current research involving human participants has been approved by the ethics committee of Shanghai First People's Hospital with written consents. The study followed the tenets of Declaration of Helsinki for the use of human subjects, and informed consents were obtained from all patients.

### Cells and cell culture

Human retinal pigment epithelial cell line ARPE-19 was routinely maintained in a 1∶1 mixture of Dulbecco's modified Eagle's medium (DMEM, Invitrogen, Carlsbad, CA) and Ham's F12 medium supplemented with 10% fetal bovine serum (FBS; Invitrogen, Carlsbad, CA). Human breast cancer cell line MCF-7 and human gingle fibroblast (HGF) cells were kindly provided by Biochemistry and Molecular biology Institute, Shanghai Jiao Tong University Affiliated First People's Hospital and Shanghai Jiao Tong University Affiliated Ninth People's Hospital, respectively. MCF-7 cells were cultured in RPMI-1640 (Invitrogen, Carlsbad, CA) with 10% FBS and insulin. HGF cells were grown in high DMEM with 5% FBS. Cells were maintained at 37°C in a humidified atmosphere with 5% CO_2_. The medium was changed twice a week. For some experiments, equal numbers of ARPE-19 cells were plated and cultivated in serum-free medium for 24 hours before stimulation with 10 ng/ml of TGF-β1 at various time points.

### Reagents and antibodies

Human recombinant TGF-β1 was purchased from HumanZyme (Chicago, US). Antibodies used in Western blot analysis and immunofluorescene were as follows: Antibodies against human E-cadherin and fibronectin were obtained from R&D systems (Minneapolis, MN). Snail antibody was purchased from Abcam Ltd (Cambridge, UK). α-SMA antibody was purchased from Sigma-Aldrich (MO, USA). ZO-1 antibody was purchased from Invitrogen (Carlsbad, CA). Puromycin, FITC-conjugated sheep anti-rabbit and FITC-conjugated sheep anti-mouse antibodies and 4′-6′-Diamidino-2-phenylindole (DAPI) were from Sigma (St Louis, MO). Sheep anti-mouse or anti-rabbit horseradish peroxidase (HRP)-labeled secondary antibodies and glyceraldehyde-3-phosphate dehydrogenase (GAPDH) antibody were from Chen Kang, Inc. (China).

### RT-PCR analysis and Real-time PCR

Total RNA was extracted from the different cell lines (TRIzol, Invitrogen-Gibco, Carlsbad, USA), according to the manufacturer's protocol. RT-PCR was performed as follows: 1 µg of total RNA was reversely transcripted using random primer (Tiangen, Shanghai, China) and reverse transcriptase (ReveTra Ace; Promega, Madison, USA), according to the manufacturer's instructions. The resulting cDNAs were employed as templates for specific PCR reactions using Taq DNA Polymerase (TAKARA, Osaka, Japan). RT-PCR of GAPDH was used as an internal control. The sequences for PCR primers were as follows: human Snail sense 5′-CAAGGAATACCTCAGCCTGG-3′; and antisense 5′-ATTCACATCCAGCACATCCA-3′; human E-cadherin sense 5′-TGGGTTATTCCTCCCATCAG-3′ and antisense 5′-TTTGTCAGGGAGCTCAGGAT-3′; human fibronectin sense 5′- GCACCAACTGACCTGAAG-3′ and antisense 5′- GCCACCATAAGTCCTGATAC-3′; human GAPDH sense 5′-GAGTCAACGGATTTGGTCGT-3′ and antisense 5′-GACAAGCTTCCCGTTCTCAG-3′. The PCR products were then subjected to electrophoresis in 1.2% agarose gel.

For Real-Time PCR analysis, quantification was performed using the 7500 Fast Real-time PCR System (Applied Biosystems, Foster, USA). The specificity of the amplification reactions was confirmed by melting curve analysis. The primer sequences used were as follows: human Snail sense 5′-CCCTCAAGATGCACATCCGAA-3′; antisense 5′-GACTCTTGGTGCTTGTGGAGCA-3′; human E-cadherin sense 5′-CGTAGCAGTGACGAATGTGGTAC-3′; antisense 5′-AACTGGAGAACCATTGTCTGTAGC-3′; Human ZO-1 sense 5′-AAGAGAAAGGTGAAACACTGCTGA-3′; antisense 5′-GGAAGACACTTGTTTTGCCAGGT-3′; human fibronectin sense 5′-CTGGAACCGGGAACCGAATATA-3′; antisense 5′-TTCTTGTCCTACATTCGGCGG-3′; human α-SMA sense 5′-AGCAGGCCAAGGGGCTATATAA-3′; antisense CGTAGCTGTCTTTTTGTCCCATT-3′; human GAPDH sense 5′-AGAAGGCTGGGGCTCATTTG-3′; antisense 5′-AGGGGCCATCCACAGTCTTC-3′. All expression data were normalized to those for GAPDH. The data were quantified with the comparative threshold cycle (*C*t) method for relative gene expression [40].

### Western blot analysis

Different time periods after treatment with 10 ng/ml TGF-β1, cells were lysed in lysis buffer (25 mM Hepes, 150 mM NaCl, 10% glycerol, 5 mM EDTA, 1% Triton X-100, 5 mM sodium orthovanadate, 50 mM NaF, 0.5 mg/ml AEBSF, and 10 µg/ml pepstatin). The samples were clarified by centrifugation at 12,000 rpm for 15 min at 4°C and boiled for 10 min with sample buffer containing 100 mM NaF. Samples were quantified and separated by 8%, 10% or 12% SDS–PAGE gel and then transferred onto a polyvinyl difluoride (PVDF) membrane (Amersham Pharmacia Biotech, Piscataway, NJ, USA). Membranes were blocked in 5% nonfat milk (diluted in Tris-buffered saline and 0.1% Tween-20) for 2 hr at room temperature. The blots were incubated with primary antibodies overnight at 4°C with rabbit anti-snail (1∶1000), anti-E-cadherin (1∶500), anti-ZO-1 (1∶1000), and mouse anti-fibronectin (1∶1000), anti-α-SMA (1∶500). This procedure was followed by incubation with sheep anti-mouse or anti-rabbit HRP-labeled secondary antibody (1∶2000) for 2 hr at room temperature. The Immunoreactive bands were visualized with chemiluminescence detection reagent (ECL, Amersham Pharmacia Biotech, Piscataway, NJ, USA).

### Examination of morphological change

The morphology of the cells was observed under an inverted phase-contrast microscope (Olympus). The photographs were taken at 100× magnifications by a digital camera.

### Immunofluorescence

The cells were seeded and cultured in 24-well chamber slides (Invitrogen, Carlsbad, USA) in serum-free medium for 24 hr and then were exposed to 10 ng/ml of recombinant TGF-β1 for the indicated time. Cells were washed three times with phosphate-buffered saline (PBS) and fixed in 4% paraformaldehyde for 10 min and washed in PBS. Slides were incubated with the indicated primary antibodies at optimal dilution for 2 hr at 37°C. After three washes, the coverslips were incubated with the appropriate fluorescent secondary antibodies diluted 1∶500 for 1 hr at room temperature. Nuclei were stained with DAPI (1∶3000). Cells were observed at timely intervals for 72 hours and pictures were obtained using a fluorescence microscopy (Carl Zeiss Inc., Oberkochen, Germany). Primary antibodies included: rabbit anti-snail (1∶100), anti-E-cadherin (1∶100), anti- ZO-1 (1∶200), and mouse monoclonal anti-fibronectin (1∶100), anti-αSMA (1∶200).

### DNA transfection

Plasmids containing Snail short hairpin RNA (shRNA) transcription unit (pSuper-Snail-shRNA) and control shRNA (pSuper-control) were kindly gifted to us by Dr. S. J. Weiss (Department of Internal Medicine, University of Michigan Comprehensive Cancer Center, Life Sciences Institute, University of Michigan, Ann Arbor, MI, USA.). Transfection of cells, at 90% confluency, was performed by Lipofectamine 2000 (Invitrogen, Carlsbad, USA) as recommended by the manufacture. Briefly, palsmids (1 µg per 24-well chamber slides) were mixed with Lipofectamine 2000 (2 µl per 24-well chamber slides) in 50 µl Opti-MEM I Reduced Serum Medium before addition to the ARPE-19 cells. Subsequently, the mixture of DNA and Lipofectamine reagent was added to the well with 1×10^6^ cells and incubated for 48 hr. Then medium was replaced with selective medium containing 1 µg/ml of Puromycin for cells transfected with pSuper-Snail-shRNA and pSuper-control, respectively. Stable transfectants were selected by picking the surviving colonies 3–4 weeks later. Single colonies were amplified and continually grew in medium containing antibiotics. Transfected cells were then collected, lysised, or treated with TGF-β1 for the following experiments.

### Migration assays

Cell migration assays were performed using Transwell chambers (8-µm pores, Costar, Conning, USA). Serum-starved transfected cells were treated by TGF-β1 (10 ng/ml) for 48 hr or untreated, and then trypsinized and counted. A total of 1×10^6^ cells were plated into the insert in 100 µl DMDM/F12 containing 0.5% FBS and allowed to migrate from upper compartment to lower compartment toward a 10% FBS gradient for 18 hr. After migration, non-migratory cells on the upper membrane surface were scrubbed off and the migratory cells attached to the bottom surface of the membrane were stained with H&E for 20 min at room temperature. The migrated cells were then enumerated. Statistical analysis was performed by two-tailed unpaired Student's t-test using the statistical software program SPSS17.0 (Chicago, IL). Data are represented as mean ± SD, with P<0.05 considered to be statistically significant.

### Epiretinal membrane specimens

Epiretinal membranes were obtained from 6 eyes undergoing vitreoretinal surgery for the treatment of rhegmatogenous retinal detachment complicated by PVR. Membranes were put into PBS (pH 7.4) during the operation and then snap frozen in liquid nitrogen or fixed in 4% paraformaldehyde. Frozen membranes were cut into small pieces and homogenized in 0.1 ml lysis buffer. The methods of protein extraction and detection were consistent with the above description.

Immunofluorescent staining was performed by a reported method [41]. In brief, epiretinal membranes were fixed in 4% paraformaldehyde at room temperature for 2 hr and washed with PBS. Rabbit polyclonal antibody against Snail (Abcam, Cambridge, UK) was applied at a 1∶200 dilution and incubated overnight at 4°C. For negative controls, PBS was used in place of the specific primary antibody. After washing with PBS, membranes were incubated with FITC-conjugated secondary antibody (Sigma, St Louis, MO) at room temperature for 1 hr. Cell nuclei were stained with DAPI (Sigma, St Louis, MO). Epiretinal membranes were flattened and observed with a fluorescence microscopy (Carl Zeiss Inc., Oberkochen, Germany).
